# Trial-unique, delayed nonmatching-to-location (TUNL): A novel, highly hippocampus-dependent automated touchscreen test of location memory and pattern separation

**DOI:** 10.1016/j.nlm.2010.07.006

**Published:** 2010-10

**Authors:** J.C. Talpos, S.M. McTighe, R. Dias, L.M. Saksida, T.J. Bussey

**Affiliations:** a Department of Experimental Psychology, Downing Street, Cambridge CB2 3EB, UK; b The Neuroscience Research Centre, Merck Sharp and Dohme Research Laboratories, Terlings Park, Harlow, Essex CM20 2QR, UK; c Behavioural and Clinical Neurosciences Institute, Department of Experimental Psychology, University of Cambridge, Downing Site, Cambridge CB2 3EB, UK

**Keywords:** High-throughput, Automated, Delayed nonmatch-to-position, Hippocampus, Spatial learning, Pattern separation

## Abstract

The hippocampus is known to be important for learning and memory, and is implicated in many neurodegenerative diseases. Accordingly many animal models of learning and memory focus on hippocampus-dependent tests of location learning and memory. These tests often use dry mazes or water mazes; however automated testing in operant chambers confers many advantages over such methods. Some automated tests of location memory, such as delayed nonmatching-to-position (DNMTP) have, however, fallen out of favor following the discovery that such tasks can be solved using mediating behaviors that can bridge the delay and reduce the requirement for memory *per se*. Furthermore some researchers report that DNMTP performance may not always require the hippocampus. Thus, in an attempt to develop a highly hippocampus-dependent automated test of location memory that elicits fewer mediating behaviors, we have developed a trial-unique nonmatching-to-location (TUNL) task, carried out in a computer-automated touchscreen testing apparatus. To test the efficacy of this assay, rats with lesions to the hippocampus, or a sham lesion control group, were tested under a variety of conditions. Both groups were able to perform well at a delay of 1 s, but the lesion group was highly impaired when tested at a 6 s delay. Moreover, animals with lesions of the hippocampus showed a greater impairment when the distance between the locations was reduced. This result indicates that TUNL can be used to investigate both memory across a delay, and spatial pattern separation (the ability to disambiguate similar spatial locations). Performance-enhancing mediating behaviors during the task were found to be minimal. Thus, the TUNL task has the potential to serve as a powerful tool for the study of the neurobiology of learning and memory.

## Research highlights

► TUNL can be used to study spatial working memory or spatial pattern separation. ► TUNL likely has fewer mediating strategies then other DNMTP tasks. ► TUNL is highly sensitive to the delay-dependent effects of hippocampal lesions.

## 1 Introduction

The hippocampus is widely acknowledged to be an important structure for learning and memory in humans, and has been implicated in many neurodegenerative diseases including Alzheimer’s disease and schizophrenia (e.g., Braak & Braak, 1997; Braak, Braak, & Bohl, 1993; Burgess, Maguire, & O’Keefe, 2002; Dickerson & Eichenbaum, 2010; Harrison, 2004; Scoville & Milner, 1957; Steen, Mull, McClure, Hamer, & Lieberman, 2006; Vargha-Khadem et al., 1997). Accordingly, many rodent models of human learning and memory focus on the hippocampus, which in the rat is known to be important for learning and memory about locations (e.g., Eichenbaum, Stewart, & Morris, 1990; Morris, Garrud, Rawlins, & O’Keefe, 1982; Olton, 1987). Rodent tests of location learning and memory often use dry mazes or water mazes, which require a large dedicated testing area and can be labor intensive. Automated tests provide an attractive alternative, as they can be carried out in operant boxes with a smaller footprint, and many animals can be tested in parallel. Furthermore such methods require less handling of animals during testing, and confer improved accuracy of task parameters. An example of a commonly used automated paradigm for studying spatial learning and memory in the rat is delayed nonmatching-to-position (DNMTP), in which the animal must remember the location (left or right) of a lever across a variable delay. However several studies have demonstrated that in this task animals were able to use ‘mediating behaviors’, such as orienting toward the to-be-correct stimulus during the delay, which could reduce the requirement for memory *per se* (e.g., Chudasama & Muir, 1997; Herremans, Hijzen, Welborn, Olivier, & Slangen, 1996). More recently, a study has been published reporting operant DNMTP to be insensitive to lesions of the hippocampus, further questioning its utility as an assay of hippocampal function (Sloan, Good, & Dunnett, 2006).

The problems with DNMTP are unfortunate because, for the reasons outlined above, an automated hippocampus-dependent location memory task would be extremely valuable. Thus, in an attempt to develop a hippocampus-dependent automated test of location memory with fewer mediating behaviors, in the present study we have developed a trial-unique nonmatching-to-location (TUNL) task. The task is set in a touchscreen testing chamber, which has been used previously to measure learning and memory in rats and mice (Brigman et al., 2008; Bussey, Muir, Everitt, & Robbins, 1997; Bussey, Muir, & Robbins, 1994; Bussey, Saksida, & Rothblat, 2001; Bussey et al., 2008; Clelland et al., 2009; McTighe, Mar, Romberg, Bussey, & Saksida, 2009; Morton, Skillings, Bussey, & Saksida, 2006). In addition, this method has been used to study hippocampus-dependent learning in a novel search task (Talpos, Dias, Bussey, & Saksida, 2008), as well as a novel test of paired-associate learning (Talpos, Winters, Dias, Saksida, & Bussey, 2009). Like DNMTP, the new TUNL task uses only two locations in any given trial, but uses multiple locations across trials. In this way, pairs of locations are repeated much less often, and the task is closer to being ‘trial-unique’ (although, like the nonspatial trial-unique matching or nonmatching-to-sample tasks [Gaffan, 1974; Mishkin & Delacour, 1975], stimuli do repeat eventually). In addition, the use of multiple locations decreases the potential to use mediating strategies. This is because the mediating responses identified by authors like Chudasama and Muir (1997) depend on the rat being able to identify, at the sample phase, which location will be the correct during the choice phase. In TUNL it is impossible to predict the correct location prior to the choice phase.

Another advantage of using multiple locations is that the distance between locations can be systematically manipulated. Kesner and colleagues (2004) have shown that the distance between locations in such tasks influences magnitude of impairment in rats with hippocampal lesions. These authors attribute this effect to a role of the hippocampus in spatial pattern separation, the ability to disambiguate overlapping spatial locations. Although touchscreen tests of pattern separation have been introduced (McTighe et al.; Clelland et al.), these tests do not allow the assessment of memory across a delay. The ability to assess spatial pattern separation in the same context as memory for locations across a variable delay could provide a single, comprehensive tool for the study of functions dependent on the hippocampus.

To test the efficacy of TUNL, animals with either lesions of the hippocampus or sham lesions were trained in TUNL and tested under multiple conditions. We predicted that lesions of the hippocampus would produce impairments in location memory when tested with a long delay, but not a short delay. Moreover we predicted that imposing delays between the sample and the choice phase would have a greater effect than that observed in other operant DNMTP tasks because of a reduction in mediating strategies. In addition, we predicted that the impairment produced by lesions of the hippocampus would interact with the distance between locations, indicating a hippocampal lesion-dependent deficit in pattern separation.

## 2 General materials and methods

### 2.1 Subjects

16 male Lister hooded rats were used for this study (Harlan, UK). They were housed four per cage and maintained on a reverse day/night light cycle (lights on from 7 pm–7 am). Rats were maintained on *ad libitum* food (standard rat chow, Purina) and water prior to surgery and during the one week following surgery. Once training began, food was restricted to maintain animals at 85–90% of normal weight; however *ad libitum* access to water was still allowed. All behavioral testing occurred during the subjects’ dark phase. All experimentation was conducted in accordance with the UK Animals (Scientific Procedures) Act, 1986.

### 2.2 Apparatus

Animals were trained in operant boxes (Med Associates, VT USA; *h* 23 cm, *w* 30 cm, 25 cm) made of a metal frame with a wall constructed of clear Perspex and metal (see Fig. 2). The floor consisted of grid bars spaced approximately 1 cm apart, situated 3 cm above a sawdust-filled waste bin. One end of the chamber was equipped with a touch-sensitive, flat-screen, LCD computer monitor (24 cm× 29 cm viewable area, Craft Data Ltd., Chesham UK). An infrared touch detection system was placed around the monitor, 4 mm from the glass of the screen, and recorded a “touch” once an object was within a few mm of the screen. This monitor was then covered with a “mask”, a piece of black Perspex (38 cm × 28 cm) with response windows cut into it (two different masks were used in this series of experiments). A spring-hinged “shelf” was attached 16 cm above the grid floor. This shelf was at a 90° angle to the mask and had a depth of 6 cm with a width of 20.5 cm. Masks were attached to the screen leaving a space of 5 mm between the mask and monitor to ensure that it would not trigger the touch-screen area. On the wall opposite from the monitor was a food magazine equipped with a light, and an infrared beam and beam detector. Above the food magazine was a house light (3 W), and a small speaker. Each operant box was housed within a sound-attenuating chamber equipped with a small fan. The boxes and monitors were controlled using IBM Netvista computers running programs written in Microsoft Visual Basic.

**Fig. 1:**
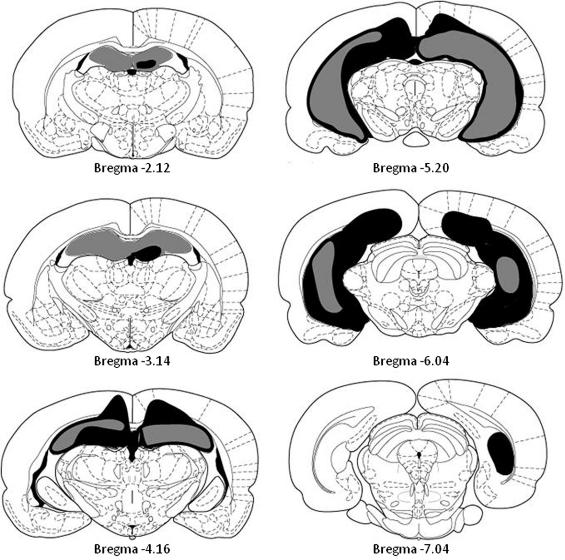
A graphical depiction of the extent of hippocampal lesions. The smallest lesion is illustrated in gray whereas the largest lesion is in black and gray.

Two different masks were used in this study. The first mask, used only during the initial training and Experiment 1 (see below), consisted of 12 square response windows arranged in three rows and four columns (only the bottom two rows were used, for a total of eight response locations). Each response window measured 4.25 cm× 4.25 cm. The lowest row was 17 cm above the floor with 1 cm separating the 1st and 2nd row, as well as the 2nd and 3rd row. Squares were equally spaced within the rows, with each row being 23 cm wide, allowing approximately 2.25 cm between columns. The second mask (used for Experiments 2–5; Fig. 1) contained three rows and seven columns for a total of 21 response windows. Again, only the bottom two rows were used for a total of 14 response locations. The total length of a row was 20 cm, whereas the total length of a column was 14 cm. All response windows were 2 cm× 2 cm equally spaced allowing a separation of 1 cm between locations.

### 2.3 Surgery

The surgical procedure used here is adapted from Ito, Everitt, and Robbins (2005). Prior to surgery, animals were randomly allocated to a lesion or a sham control group. All animals were anaesthetized with Avertin during the course of surgery. Once anaesthetized, rats were placed in a stereotaxic frame with the incisor bar set at 3.3 mm below the interaural line (Kopf, USA). Small holes were drilled into the skull above the injection sites. Lesioned animals then had a bevel-tipped micro-syringe lowered into the hippocampus to deliver *N*-Methyl-d-Aspartic acid (0.09 M in sterile PBS, Sigma Aldrich, UK) into the hippocampus (see Table 1 for more detail). Anterior–posterior, as well as medial–lateral coordinates were calculated relative to bregma, whereas dorsal–ventral coordinates were calculated relative to dura. Immediately after surgery animals were placed in heated chambers in a darkened room and allowed to recover with free access to food and water. Diazepam (5 mg/ml, volume 0.1 ml/kg, i.m.) was administered if fitting occurred during, or immediately after surgery.

### 2.4 Histology

When all behavioral testing was completed, animals were anaesthetized with Dolethal (2 ml, i.p.) and transcardially perfused with 100 ml of PBS, followed by 250 ml of 4% paraformaldehyde. Brains were then removed and further fixed in paraformaldehyde at 4 °C for 24 h. Prior to cutting, samples were immersed in 20% sucrose in PBS for 24 h. Sixty micrometer sections were cut using a cryostat, and every fifth section was mounted on a gelatin-coated glass slide, and then stained with cresyl violet. When completed, lesion and control brains were examined under a light microscope to determine the extent of damage.

The largest and smallest lesions are displayed in Fig. 1. Subject 1 showed partial sparing of the posterior ventral CA1 region (AP −6.0). Subject 2 had sparing to the most posterior CA1 region (AP −6.3) as well as substantial damage to the corpus callosum. Subject three had sparing of left posterior and right posterior ventral CA1 region, as well as slight damage to the corpus callosum. Subject four (illustrated) showed complete destruction of the hippocampus. However, substantial damage was also seen to the corpus callosum (9 of 29 sections showed at least some damage) and the retrosplenial cortex. Subject 5 had bilateral sparing of the ventral posterior CA1 region (AP −6.0), increasing to total sparing of the most ventral portions of the CA1 region, as well as some damage to the corpus callosum. Subject 6 had sparing to the CA1 region along the sagittal line, beginning dorsally, and spreading ventrally (AP −3.8). However sparing never included ventral portions of the CA1 region. Subject 7 exhibited partial right anterior CA3 sparing throughout the whole brain, as well as minor bilateral sparing along the dorsal sagittal line. This sparing occurred into the right most posterior CA1 region. Subject 8 (illustrated) showed sparing to the left anterior CA3 region as well as the right dorsal and ventral CA1 region. Subject 1 from the sham group was excluded from the study as it showed abnormalities within the hippocampus. This cohort of animals was used for Experiments 1–4.

### 2.5 Training

#### 2.5.1 Pre-training

Prior to training, animals were placed in the operant chambers for 20 min to habituate to the environment. Food pellets were placed in the food receptacle as well as on the shelf and mask attached to the touchscreen. During this time the touchscreen was not activated. After habituation, animals were trained to respond to a tone for a food. This was accomplished by delivering a 0.5 s tone, followed by a food pellet, every 30 s. During this time, white squares were presented in all of the response windows. If an animal touched the monitor it was rewarded with three food pellets, and the beginning of the next trial was initiated. However, no response also resulted in the tone and a one-pellet reward after 30 s. This procedure was followed for one session (100 trials) to ensure the rat had learned the association between a tone and food. Next, rats were required to touch any area of the monitor while all squares were displayed to earn a reward. The screen remained active until a response occurred. Once a response occurred a tone was sounded, a food pellet was delivered, and the touchscreen was deactivated. The next trial began 5 s after the pellet was collected. A session was complete when either 30 min passed, or after the rat had completed 100 trials. Animals were trained in this fashion until they successfully completed 100 trials within 30 min. This typically took 3 sessions. A final stage of training was used to ensure that animals did not develop a bias to one area of the screen. In this stage one of the eight response locations would be randomly illuminated and the rat was required to poke at this location to earn a reward. Pokes at other locations were not punished or rewarded. When successful, a reward pellet was delivered. Eating this food pellet would trigger the inter trial interval (5 s) and the next trial, with a new location, would begin after a nose poke into the food magazine. This was repeated until each rat could complete 100 trials in 1 h.

#### 2.5.2 Training on TUNL

After all animals had undergone pre-training they were trained on the full version of TUNL using a 6 s delay, and a 10 s ITI. Once in the testing chamber, rats were required to nose poke into the food receptacle to start the session and a trial. This triggered the sample stimulus to appear in any of 8 locations (14 in later trials). Once a response was recorded at this location the stimulus ceased to be illuminated, ending the sample phase. After the delay period the food receptacle was illuminated. When illuminated, that rat was required to poke in the receptacle to begin the choice phase of the trial. During this phase of the trial the sample location was again illuminated (S−) along with a new location that served as the S+. If the animal correctly selected the S+ then the stimuli would be removed from the screen, a tone would sound in conjunction with the delivery of a reward pellet and the activation of food receptacle light. Nose-poking into the receptacle to eat the reward pellet would result in deactivation of the light and the initiation of the ITI. Once the ITI period had passed the food receptacle would again be illuminated to signal that a poke was required to begin the next trial. If the trial ended in an incorrect response then the stimuli would be removed from the screen, and the house light would be extinguished for 5 s. After this timeout the ITI would begin. Once the ITI had passed, the food receptacle light was activated to signal that a nose poke was needed to begin the next trial. Once this poke had occurred then the ITI would begin and the next trial would be identical to the previous in that the sample and choice would be in the same locations. These “correction” trials were used to protect against the development of bias to any particular location. Activation of the sample location was rewarded with a single pellet on 1/3rd of the trials. A session was considered complete after either an hour had passed, or the rat had completed 64 trials, whichever occurred first.

### 2.6 Results

Unless otherwise indicated, repeated-measures ANOVAs were used to analyze data and post hoc analysis was performed using a Student Newman-Keuls test (SNK). When appropriate, *t*-tests with the mean set at 50% were used to compare accuracy against chance. The percentage of correctly completed trials (correct trials/total completed trials × 100; excluding correction trials) was used as the dependent variable. The independent variables were lesion and, where appropriate, trial block, session, and S+/S− separation. A summary of statistical tests used by experiment can be seen in Table 2.

## 3 Experiment 1: initial acquisition of TUNL

### 3.1 Behavioral testing methods

Subjects were tested on a trial-unique version of the task using a mask with 3 rows of 4 response locations and a delay of 6 s. However only the bottom 2 rows were used for a total of 8 locations. Subjects were run on this version of the task for 20 days. Trials were divided into three groups based on separation between the S+ and S−: maximum, medium, and adjacent. In the adjacent separation condition the S+ and S− were next to each other, in the medium separation condition the S+ and S− were separated by one location, and by 2 locations in the maximum separation condition.

### 3.2 Results

Prior to analysis, acquisition data were averaged into 4 blocks of 5 sessions each. A main effect of block (*F*(3, 39) = 3.82, *P* = 0.017) and lesion (*F*(1, 13) = 19.48, *P* = 0.001) was seen. The sham group was found to make significantly fewer errors than the hippocampus-lesioned group, as indicated in Fig. 3. No significant interaction was seen between testing block and lesion (*F*(3, 39) = 2.36, *P* = 0.086). Importantly, the lesion group did perform above chance (*t*(1, 7) = 2.98, *p* = 0.021). The final block of data was analyzed to investigate the effect of stimulus separation on performance (Fig. 4). A main effect of lesion was seen (*F*(1, 13) = 19.13, *P* < 0.001), while separation failed to reach significance (*F*(2, 26) = 2.35, *P* = 0.11). However an interaction was seen between lesion and separation (*F*(2, 26) = 5.06, *P* = 0.014; Fig. 4). Post hoc analysis indicated significant effects of lesion on accuracy at medium (SNK, *p* = 0.023) and maximum separation (SNK, *p* = 0.041); where again the shams were found to make fewer errors.

### 3.3 Discussion

The main result from this experiment is that lesions of the hippocampus cause an impairment in acquisition of TUNL. Acquisition in this case was tested under conditions of a 6 s delay. The advantage of a task like TUNL, however, is that manipulations can be made on a performance baseline; behavioral manipulations include variable delays, as well as variable separations. This allows for the detection of delay-dependent and separation-dependent impairments, both of which we predicted would be observed following hippocampal lesions. Accordingly in Experiment 2 we tested performance in TUNL at short (6 s), and very short (1 s) delays under various separation conditions.

## 4 Experiment 2: the effects of changes in delay and separation on accuracy

### 4.1 Behavioral testing methods

After initial acquisition subjects were tested using a mask that had 3 rows of 7 columns, but only the bottom two rows were used for a total of 14 possible locations. Rats were tested in two, two session blocks, at each of four separation and delay combinations. The delays used were 1 s or 6 s, and the separations were either minimum (1 response area separated the S+ and S−), or maximum (5 response areas separated the S+ and S−). These were combined in a 2 × 2 design.

### 4.2 Results

#### 4.2.1 6 s delay and maximum separation between stimuli

A significant effect of lesion (Fig. 5) was seen (*F*(1, 13) = 27.48, *P* = 0.0002. No significant effect of block (*F*(3, 39) = 0.58, *P* = 0.63) or interaction between block and lesion was seen (*F*(3, 39) = 0.48, *P* = 0.70).

#### 4.2.2 6 s delay and minimum separation between stimuli

After 8 test sessions (4 blocks of two sessions) in this condition a significant effect of lesion was seen (*F*(1, 13) = 6.26, *P* = 0.026) indicating that the shams made significantly fewer errors than did the lesion group (Fig. 5). This effect was numerically smaller than that previously seen, due to the sham group performing much more poorly under conditions of minimum separation. No significant effect of block (*F*(3, 39) = 0.37, or block by session interaction (*F*(3, 39) = 0.94, *P* = 0.43) was seen.

#### 4.3.1 1 s delay and maximum separation between stimuli

After 8 test sessions (4 blocks of two sessions) a significant effect of block (*F*(1, 13) = 4.64, *P* = 0.0072), but not lesion (*F*(1, 13) = 4.07, *P* = 0.065) was seen. Accuracy improved with passing blocks. However no interaction between lesion and block was seen (*F*(3, 39) = 1.56, *P* = 0.21; Fig. 5).

#### 4.3.2 1 s delay and minimum separation between stimuli

No significant effect of lesion (*F*(1, 13) = 0.081, *P* = 0.38), block (*F*(3, 39) = 0.17, *P* = 0.92), or block by session interaction (*F*(3, 39) = 0.65, *P* = 0.71) was seen as illustrated in Fig. 5.

### 4.4 Discussion

Animals with lesions of the hippocampus, or sham control lesions, were tested in TUNL with multiple combinations of delay and separation. Initially, both groups were tested with a maximum separation and a 6 s delay. In this condition the sham group achieved an accuracy of approximately 80% correct compared to the lesion group’s approximately 60% correct.

Subjects were tested again under a 6 s delay, but this time under a minimum separation condition. As predicted both groups showed low levels of accuracy. Next, both groups were tested with a 1 s delay under conditions of maximum separation. A marked increase was seen in performance of both groups. In this instance the sham group reached over 80% correct and no significant difference was seen between the lesion and sham groups. These data indicate that rats with hippocampal lesions can, under baseline conditions, perform as well as controls, but they are very vulnerable to the effects of delay. Furthermore both sham and lesion groups are greatly affected when the stimulus separation is reduced. Last, to confirm this conclusion, subjects were tested with a 1 s delay and a minimum stimulus separation. As predicted, performance of both groups dropped to chance, and an effect of lesion was no longer seen (sham performance being at floor).

In the present experiment the presentation of the four conditions was not counter-balanced to control for effects of order of testing. In Experiment 3, therefore, we tested variable delays and separations using a counter-balanced design.

## 5 Experiment 3: the effects of counter-balanced delays with mixed separations on accuracy

### 5.1 Behavioral testing method

The procedure used here was the same as during acquisition of the task, with two changes. First, as in Experiment 2, only the bottom two rows in a 3-row by 7-column mask were used. Also, trials where the S+ was adjacent to the S− were not included as results from earlier experiments indicated that control rats did not perform above chance under these conditions. Separations were now calculated as follows: the distance from one location to an adjacent location to the left or right, or above or below, was defined as 1. Thus, distances between locations could be computed as Euclidean distances, for example, the distance from one location to an adjacent location diagonal to that location would be √2. For analysis with separation as a factor distances less than 3 were considered minimum, distances of 3 to less than 6 were considered medium, and distances of 6 and greater were considered maximum. The reason for this grouping was to allow suitable numbers of trials in each condition to performing meaningful statistical analysis. Multiple separations were used within one testing session while delay was kept constant within a session but varied between sessions.

### 5.2 Results

Main effects were seen of lesion (*F*(1, 11) = 6.81, *P* = 0.024), delay (*F*(1, 11) = 12.88, *P* = 0.004), and separation (*F*(1, 11) = 7.63, *P* = 0.0030) (see Fig. 6). A significant interaction was seen between delay and lesion (*F*(1, 11) = 7.16, *P* = 0.021), but not separation and lesion (*F*(2, 22) = 0.55, *P* = 0.58), or delay and separation (*F*(2, 22) = 2.02, *P* = 0.16). However, a significant 3-way interaction was seen between delay, separation, and lesion (*F*(2, 22) = 5.15, *P* = 0.014). Therefore, additional ANOVAs were done to examine the effects of delay and lesion at medium and maximum separation. Minimum separation conditions were not included in the additional analysis as performance was consistently near 50%, and not statistically above chance. A two-way ANOVA for delay and lesion (maximum separation was used as it best allowed a detection of the effect of delay) indicated a significant effect of delay (*F*(1, 11) = 6.70, *P* = 0.025) and interaction between delay and lesion (*F*(1, 11) = 10.37, *P* = 0.0081). Moreover the effect of lesion approached, but failed to reach significance (*F*(1, 11) = 3.89, *P* = 0.074; Fig. 7). Post hoc analysis (Dunnett’s was used in this instance as the specific difference between the 6 s lesion group compared to 6 s sham, 1 s sham, and 1 s lesion was the only comparison of interest) indicated that the performance of the lesion group under the 6 s condition was significantly different from that of all other groups including the sham group under the 6 s condition (*F*(1, 11) = 7.02, *P* = 0.023) and the lesion group under the 1 s condition (*F*(1, 11) = 4.95, *P* = 0.048). Last, a two-way ANOVA with lesion and separation as the dependent variables (1 s delay) indicated a main effect of separation (*F*(2, 22) = 9.55, *P* = 0.0010; Fig. 8). However no main effect of lesion (*F*(1, 11) = 2.29, *P* = 0.16), or interaction between separation and lesion (*F*(2, 22) = 1.11, *P* = 0.35) was seen.

### 5.3 Discussion

The data presented here confirm the findings from Experiment 2 – that when tested in TUNL, rats with lesions of the hippocampus are extremely sensitive to delay. Striking evidence for this is seen by comparing performance of the sham and lesion group at 1 and 6 s delays under the maximum separation condition. At a 1 s delay it appears that an intact hippocampus is not needed for accurate performance on this task. However, increasing the delay between the sample and choice phases to just 6 s is enough to cause a massive impairment in the performance of the hippocampal lesion group, even at the highest stimulus separation. We suspect this is because mediating strategies are not as effective in a trial-unique paradigm as compared to repeating-items DNMTP (this idea is tested further in Experiment 5).

Other work has suggested that the hippocampus is necessary for successful spatial pattern separation (Clelland et al., 2009; Long & Kesner, 1996; McTighe et al., 2009). However, we have yet to see statistically significant evidence for this effect here (*P* values approach, but fail to reach significance), although Fig. 7 clearly indicates a tendency for the hippocampal lesion group to be impaired under the lower, but not the higher separation conditions. Accordingly, the next phase of this study was designed specifically to test for an interaction between separation and lesion. To do this, animals were tested with a 1 s delay at multiple separations (separation was held constant within a session but varied between sessions in a counter-balanced fashion). In this way animals were tested under conditions ideal to detect an effect of hippocampal lesions on pattern separation, while the increased number of trials dedicated to each separation decreased variation in accuracy. The inclusion of a wide range of separations would also ensure that critical separations, where the groups may diverge, would be included.

## 6 Experiment 4: an investigation of the interaction between separation and lesion

### 6.1 Behavioral testing method

This experiment was designed to test explicitly for an interaction between lesions of the hippocampus and the distance between the two locations. The testing apparatus was the same as in the Experiments 2 and 3. Animals were tested with a 1 s delay at 5 different levels of separation. Levels of separation were presented in a counter-balanced fashion, with just one level being tested in any given session.

### 6.2 Results

After 15 test sessions at 5 different separations, mean performance was calculated for each separation. The resulting repeated-measures ANOVA indicated a main effect of lesion (*F*(1, 13) = 6.98, *P* = 0.020) and separation (*F*(4, 52) = 42.57, *P* < 0.0001). Sham lesioned rats again performed better at larger rather then smaller separations. Furthermore, as predicted, an interaction was seen between separation and lesion (*F*(4, 52) = 3.00, *P* = 0.026). Post hoc analysis (SNK within subject, Fig. 9) indicated significant differences at separations of 4 (*P* = 0.004) and 2 (*P* = 0.049).

### 6.3 Discussion

As in previous experiments, main effects of lesion and separation were seen. In this instance analysis also indicated an interaction between lesion and separation. Both groups were sensitive to the effects of separation, but the lesion group more so than the control group. As the separation between locations decreased, a greater decrease in performance was seen in the lesion group than in the control group, an effect similar to that reported by Kesner and colleagues. In their study using a maze-based procedure, decreasing the separation between the S+ and S− caused a decrease in accuracy (Gilbert, Kesner, & DeCoteau, 1998; Kirwan, Gilbert, & Kesner, 2005). Lesions of the hippocampus exacerbated this effect (Gilbert & Kesner, 2006; Gilbert et al., 1998; Rolls & Kesner, 2006). Kesner and colleagues interpret this finding as indicating a crucial role for the hippocampus in spatial pattern separation. Thus, TUNL provides an automated method with which to assess simultaneously memory across a delay, and spatial pattern separation.

## 7 Experiment 5: analysis of putative performance-enhancing mediating behaviors

This final experiment was performed to evaluate the ability of rats to use mediating behaviors such as those shown in DNMTP to bridge the delay and thus reduce the memory load of the task (Chudasama & Muir, 1997; Herremans et al., 1996). It was thought that, in contrast to the repeated-items nature of the 2-lever DNMTP paradigm, the more trial-unique nature of TUNL would make it difficult for animals to develop such behaviors, because the correct location cannot be determined until the choice phase of the task. The elimination of orienting toward the to-be-correct location does not guarantee, however, that rats could not possibly find other mediating behaviors with which to enhance their performance on the task. Thus, we sought to test stringently whether we could identify any such behaviors. We videotaped the animals during a session in which the parameters were set in a way that we thought would likely encourage mediating behaviors. Trials with the locations at the farthest separation were used, thus increasing the utility of left or right-directed orienting behaviors. This non-trial-unique condition is reminiscent of the 2-lever operant DNMTP task. We also used a delay of 6 s, the delay at which hippocampus-lesioned rats performed at chance. We identified 18 candidate behaviors, and analyzed whether the performance of any of these behaviors led to increases in performance on the task.

### 7.1 Methods

#### 7.1.1 Apparatus

For this experiment, a webcam (Quickcam; Logitech, Switzerland) was positioned inside the sound attenuating box above the operant chamber. The camera was attached to a fish-eye lens and oriented to capture video of the whole operant chamber, as well as the screen. The camera was connected to a computer via a USB cable, and videos were recorded onto the computer using Logitech Webcam Software (Logitech, Switzerland).

#### 7.1.2 Subjects

12 naive Lister Hooded rats were used for this phase of the study (Harlan UK; 250–270 g at the start of the experiment). They were housed in pairs on a 12hr/12hr reverse day/night cycle (lights on 7 pm). Rats were allowed to habituate for one week prior to being place on a restricted feeding regime (85–90% of free feed weight). Access to water was *ad libitum*. All experimentation was conducted in accordance with the UK Animals (Scientific Procedures) Act, 1986.

### 7.2 Behavioral procedures

#### 7.2.1 Training on TUNL

TUNL training was carried out using a similar procedure to that in Experiments 1–4. Animals were trained using a 20 s ITI and a 6 s delay. Correction trials were included as in previous experiments.

#### 7.2.2 Testing

After reaching a steady state of responding on the TUNL task, the behavior of each rat was recorded during a single testing session (48 trials). During this session, rats were tested exclusively at the largest separations, equivalent to separations 5 and 6 in Experiment 4. This was conducted using a 20 s ITI and a 6 s delay. Correction trials were administered but were not included in the analysis.

#### 7.2.3 Data collection

In addition to the computerized collection of data as previously described, behavior during testing was also recorded using a webcam and recorded onto a computer (Dell, UK). An experimenter then scored the videos by hand. Unlike previous papers on the issue of mediating strategies in DNMTP (e.g., Chudasama & Muir, 1997), we were unable to identify any behaviors a priori that appeared to be mediating strategies. Instead, we identified 18 candidate behaviors, including left and right orienting behaviors and screen touches during the delay, that might on further analysis prove to be beneficial to the animals’ accuracy on test. For each trial in the session the experimenter recorded whether any of these 18 candidate behaviors were present. For all behaviors, “left” and “right” are defined as relative to the box, (and therefore the stimuli on the screen) rather than the rat. The 18 behaviors were: **body turn** right or left – rat turns its whole body to one side; **head turn** right or left – rat turns just its head to one side; **orienting forwards** right or left – while positioned anywhere in the box, the rat orients itself towards the screen for a period of 1s or more; **orienting backwards** right or left – while positioned anywhere in the box, the rat orients itself towards the back of the box (away from the screen) for a period of 1s or more; **orienting sideways** right or left – while positioned anywhere in the box, the rat orients itself sideways for a period of 1s or more; **orienting in the magazine** right or left – the rat orients with its head in the food magazine for a period of 1s or more; **back and forth** right or left – the rat moves back and forth between one side of the screen and the food magazine; **waiting** right or left – the rat waits at an approximate distance of 5 cm or less from the screen for 1s or more (distinguished from orienting by the rat being very close to the screen, either rearing with forepaws touching the shelf, or head looking up at the screen); **screen-touch, sample** – the rat touches the screen at the sample square; **screen-touch, other** – the rat touches any other square on the screen.

#### 7.2.4 Analysis

To determine which behaviors might help bridge the delay and reduce the requirement for memory, it was necessary to calculate the relative benefit (or cost), in terms of performance on the task, the animal received from each behavior. To do this, we calculated a *benefit score* for each behavior. First, we determined the proportion correct from trials in which the behavior was *not* present. This value was then multiplied by the number of trials on which the animal exhibited the behavior, to give the “expected number” of correct trials that would be obtained if the animal had never used the behavior. This expected number correct was then subtracted from the actual number of trials the animal got correct whilst performing the behavior – a positive number indicates a benefit of the behavior and a negative number indicates a cost. This benefit/cost was then expressed as a percentage of the total trials completed by each rat. This percent benefit/cost was then averaged across rats (see Eq. (1)). The means of the group of 12 rats for each behavior is shown in Fig. 10.

(1) $$\overset{¯}{x}=\frac{1}{n}\overset{n}{\underset{i=1}{\sum }}\left[\frac{1}{e_{i}}\left(a_{i}-\frac{b_{i}c_{i}}{d_{i}}\right)\right]$$

where $\overset{¯}{x}$ = mean of all observations, *n* = number of rats, *a_i_* = number of trials correct in the presence of the behavior for each rat, *b_i_* = number of trials correct in the absence of the behavior for each rat, *c_i_* = total number of trials conducted in the presence of the behavior for each rat, *d_i_* = total number of trials conducted in the absence of the behavior for each rat, *e_i_* = grand total number of trials for each rat.

Here the benefit is expressed as a proportion; in the text it is referred to in percentages for ease of description.

### 7.3 Results

#### 7.3.1 Performance

The average number of trials completed was 45.2 (±2.0). Of the 12 animals, only 3 did not complete 48 trials, completing 25, 41 and 44 trials. Rats’ mean performance on the videotaped sessions was 72.7% correct, with an SEM. of ± 2.6%.

#### 7.3.2 Analysis of candidate mediating behaviors

The benefits and costs of each candidate mediating behavior as calculated in Eq. (1) are shown in Fig. 10. Bonferroni-corrected *t*-tests revealed that none of the 18 candidate behaviors identified significantly improved (or impaired) performance on the task. If we inflate alpha by performing 18 non-corrected *t*-tests, 3 behaviors appear to generate significant benefits/costs; they are **body turn right** (*p* = 0.020), **orienting sideways left** (*p* = 0.027), and **screen-touch sample** (*p* = 0.008). By this analysis **body turn right** produced a decrease in percent correct, whereas **orienting sideways left** and **screen-touch sample** produced improvements. If these comparisons can be taken as evidence for improvement due to the behavior, the numerical improvements were very small: 0.68 ± 0.22% for **orienting sideways left**, and 1.65 ± 0.55% for **screen-touch sample**.

### 7.4 Discussion

A long-standing criticism of operant, two-lever DNMTP paradigms has been the possible use of mediating behaviors that can bridge a delay, thereby reducing the demand on memory *per se*. For example, Chudasama and Muir (1997) report that rats can orient towards the location (lever) that will be correct on choice. TUNL was designed in part to minimize such strategies by using many locations per session so that the location that will be correct on choice cannot be determined at the sample phase. The elimination of orienting toward the to-be-correct location does not guarantee, however, that rats could not possibly find other mediating behaviors with which to enhance their performance on the task (for instance perseverating at the sample location). Thus, in the manner of Chudasama and Muir, we sought to test stringently whether we could identify any behaviors that might be used to mediate during the delay. First we videotaped the animals during a session in which the parameters were set in a way that we thought would likely encourage mediating behaviors. Trials with the locations at the farthest separation were used, thus increasing the utility of left or right-directed orienting behaviors. This non-trial-unique condition is reminiscent of the 2-lever operant DNMTP task. We used a delay of 6 s, the longest tested in this study and the delay at which hippocampus-lesioned rats performed at chance. Of the 18 candidate behaviors identified, only two showed a significant benefit, and only when uncorrected *t*-tests were used (increasing the likelihood of a false positive result). If these comparisons can be taken as evidence for improvement due to the behavior, the numerical improvements were small: **0.68%** for **orienting sideways left**, and **1.65%** for **screen-touch sample**. Regarding **orienting sideways left**, it is difficult to see how the ‘left’ version of a behavior could improve performance whereas the ‘right’ version did not (there were an equal number of left and right correct trials, and the (non-significant) improvement generated by the ‘right’ version of this behavior (**orienting sideways right**) was close to zero (**0.09%**)). Regarding **screen-touch sample**, however, it is conceivable that some rats could orient towards the location in which the sample was presented in order then to *avoid* that location on choice and choose whatever other location was presented. If this is what the rats are doing, however, the behavior still only earns them an average performance increase of under 2% (and of course this ‘improvement’ was not significant when we correct for the number of comparisons). Absence of evidence cannot be taken as evidence for absence. It is possible that some subtle performance-enhancing behavior may have eluded our scrutiny – our analysis of candidate behaviors was thorough, and these data indicate that rats use performance-enhancing mediated strategies in TUNL very little, if at all. Finally, even if such behaviors are being used, they were not used to benefit the rats with hippocampus lesions; these rats performed at chance level under the same conditions under which we analyzed for mediating behaviors. It is conceivable that the lesion could impair the mediating strategy, thus producing what looks like an impairment in memory, and indeed there is some evidence for this idea (Chudasama & Muir, 1997). The drastic drop in performance caused by the hippocampal lesions on TUNL, from about 80% correct down to about 50%, cannot be easily explained by the lesion knocking out a mediating behavior that improves performance only by less than 2%. We thus conclude that TUNL is highly hippocampus-dependent, likely more delay dependent then DNMTP, probably due at least in part to trial-unique training which discourages mediating behaviors.

## 8 General discussion

In the present study we developed and tested a trial-unique nonmatching-to-location (TUNL) test of location memory and pattern separation. The task was shown to be remarkably sensitive to hippocampal damage. Excitotoxic lesions of the hippocampus reduced performance to near-chance levels at a 6 s delay, even in the easiest separation condition. In addition, when tested in the easiest delay condition (1 s), rats with hippocampal lesions were impaired at the small, but not the large separations, indicating that TUNL can detect impairments in spatial pattern separation. Furthermore, analysis of potential mediating responses found little evidence for behaviors that were used to reduce memory load by bridging the delay.

**Fig. 2:**
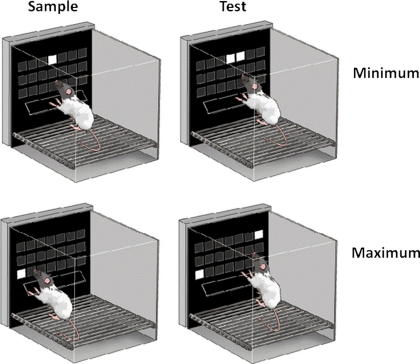
A depiction of the testing apparatus during a low separation trial (upper panes) and a high separation trial (lower panes). Both the choice (left panes) and sample phase (right panes) are shown.

**Fig. 3:**
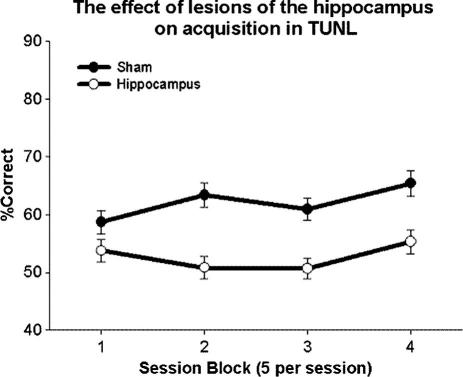
Hippocampal lesions impair acquisition of TUNL. Each block represents 5 testing sessions. Error bars represent 1 SEM.

**Fig. 4:**
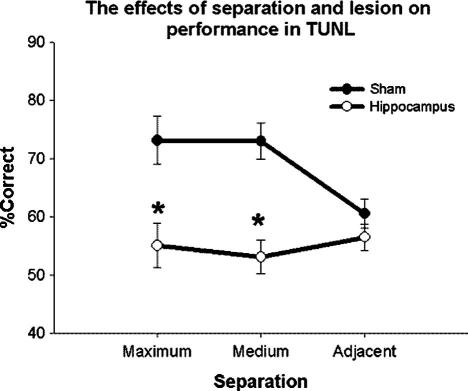
The effects of hippocampal lesions and separation on the final trial block of TUNL (6 s delay). A significant effect of lesion and interaction between session and separation was seen. Error bars represent 1 SEM. ^∗^*P* > 0.05.

**Fig. 5:**
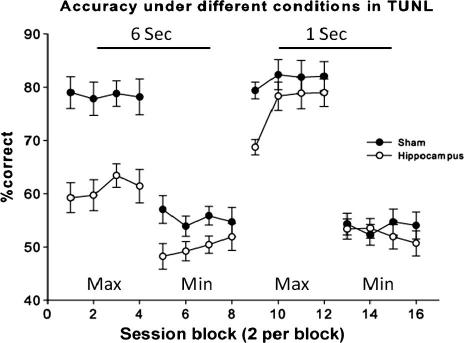
Performance on TUNL was evaluated under several different testing conditions. Each block refers to two testing sessions. Session blocks 1–4 represent performance with a 6 s delay and maximum separation, a significant effect of lesion was seen. Session blocks 5–8 represent performance with a 6 s delay and minimum separation. A significant effect of lesion was seen. Session blocks 9–12 represent a 1 s delay with a maximum separation. No effect of lesion was seen. Session blocks 13–16 represent testing with a 1 s delay and minimum separation. No effect of lesion was seen. Throughout the figure error bars represent 1 SEM.

**Fig. 6:**
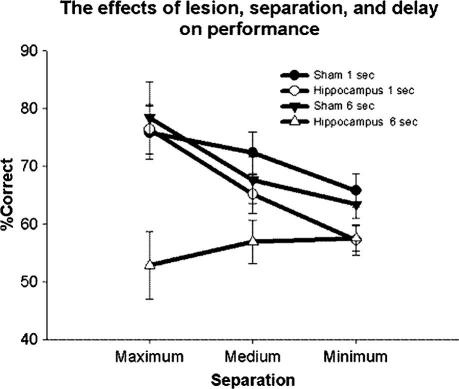
Under counter-balanced conditions, the effects of lesion, delay, and separation are compared. Error bars represent 1 SEM.

**Fig. 7:**
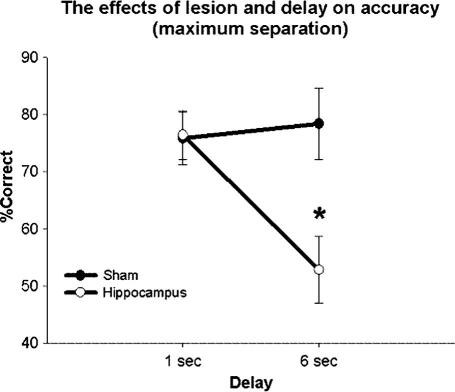
The interaction between delay and lesion under a maximum separation testing condition is illustrated. There was a main effect of delay, and an interaction between delay and lesion. Error bars represent 1 SEM. ^∗^*P* > 0.05.

**Fig. 8:**
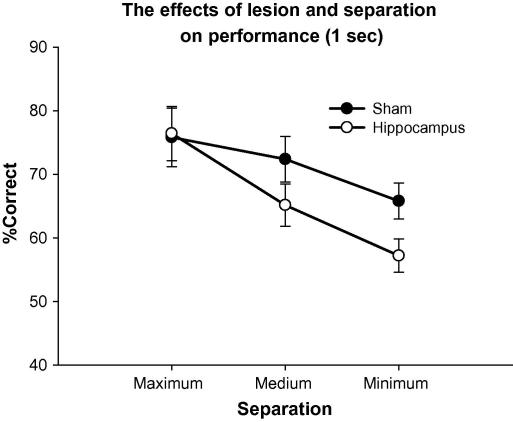
The main effect of separation with a 1 s delay is illustrated. Error bars represent 1 SEM.

**Fig. 9:**
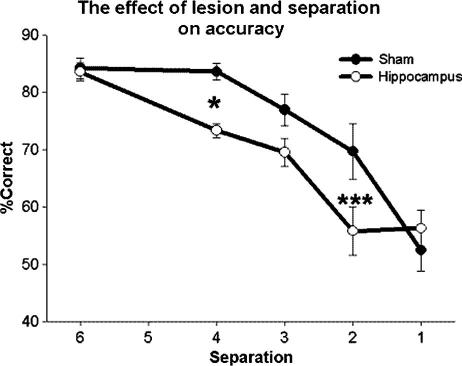
The main effect of lesion and separation when tested at a 1 s delay and multiple distances is illustrated. The *x*-axis displays the separation of the S+ and S− during the choice phase of the trial. Error bars represent 1 SEM. ^∗^*P* > 0.05, ^∗∗∗^*P* > 0.001.

**Fig. 10:**
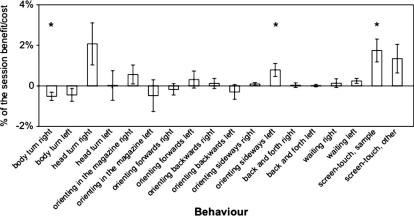
Analysis of the efficacy of putative mediating strategies. The estimated change in accuracy (across rats) associated with each behavior, if that behavior was used for all trials completed, compared to the observed performance. When calculating the observed performance, any trials were the behavior of interest was observed, were removed. Inclusion of these trials would have artificially decreased the effect of any mediating strategies.

TUNL appears to be more sensitive to hippocampal dysfunction than other automated location memory tasks such as operant DNMTP, as suggested by the discrepancy between accuracy levels in our task and those reported in other paradigms. In the present study we found performance was highly impaired in lesioned animals, a delay of only 6 s causing near chance-level performance (Fig. 2). This is in contrast to the results seen by Chudasama and Muir (1997) who report that with an 8 s delay the sham lesion group performed at about 85% correct while animals with fornix lesions performed at about 75% correct. Although a statistical comparison between the groups in these two studies is not appropriate, the hippocampal-lesioned animals appear to perform much worse on TUNL than do fornix-lesioned animals on DNMTP. This discrepancy could, of course, be due at least in part to the different methods of inducing hippocampal dysfunction in the two studies. This seems unlikely, however, as if anything fornix lesions have a greater effect than hippocampal lesions in spatial tasks, and the resulting dysfunction is not limited to the hippocampus (Aggleton, Keith, & Sahgal, 1991;Vann, Brown, Erichsen, & Aggleton, 2000). Instead, the striking impairments seen in TUNL could have been caused by increased cognitive demand due to the task being more trial-unique, using a number of locations on the screen rather than only two, as in DNMTP. One effect of using multiple locations is that unlike the situation in tasks such as two-lever DNMTP, the rat cannot know in advance which location will be correct upon choice. The rat cannot use certain behaviors, such as orienting toward the to-be-correct stimulus, which have been observed in DNMTP to bridge the delay (Chudasama & Muir, 1997), likely reducing the memory demands of the task. The minimal use of such mediating behaviors was confirmed in our analyses of potential mediating behaviors in Experiment 5. The use of multiple locations confers other advantages, as well. For example, the use of multiple locations and therefore numerous trial-types allows the analysis of behavior under conditions of variable distances between the two locations. A number of studies indicate that altering the distance between locations can have a detrimental effect on animals with hippocampal dysfunction(Gilbert & Kesner, 2002; Gilbert et al., 1998; Goodrich-Hunsaker, Hunsaker, & Kesner, 2005; Kesner et al., 2004; McDonald & White, 1995; McTighe et al., 2009; White, 2004). These authors interpret these findings in terms of spatial pattern separation, the ability to disambiguate spatial representations, allowing discrimination of locations that have overlapping spatial elements. This function is thought to be mediated by the dentate gyrus/CA3 complex more specifically (Bakker, Kirwan, Miller, & Stark, 2008; Gilbert & Kesner, 2006; Kesner et al., 2004, McHugh et al., 2007). Furthermore, more recent evidence suggests that hippocampal neurogenesis may be particularly important for pattern separation (Clelland et al., 2009).

Although the pattern of data reported by these authors agrees entirely with that found in the present study, it is worth noting the differences. Gilbert et al. (1998), for example, reported impairments after hippocampus lesions when separations were reduced to 82.5 cm, whereas in the present study we observed such effects only at a fraction of that distance. An obvious difference between the two studies is the apparatus used. Gilbert et al. (1998) used an open field several times larger than the operant box used in the present study, and so the spatial cues available to the animals would have been relatively distal to the locations the rat had to discriminate. In contrast the cues available to the animal in the touchscreen method must be located within the operant chamber, a short distance from the locations to be discriminated on the computer screen. It is easy to imagine that locations might be more difficult to discriminate when the cues on which the discrimination are based are farther away from those locations, and thus might put a higher demand on pattern separation. This is an idea that could be tested empirically.

To conclude, TUNL appears to be a comprehensive test of hippocampal-dependent spatial cognition with greatly reduced opportunity for the development of mediating strategies. Its real strength, however, lies in the fact that it can be tested in the same apparatus as other diverse tests of cognitive function such as visual discrimination and reversal learning (e.g., Bussey et al., 1997, 2001; Brigman et al., 2008; Brigman, Ihne, Saksida, Bussey, & Holmes, 2009), spatial search tasks (Talpos et al., 2008), visuo-motor conditional spatial discriminations (e.g., Bussey, Muir, Everitt, & Robbins, 1996), spatial reversal learning (McTighe et al., 2009), and paired-associates learning (Talpos et al., 2009). When combined with these tasks, it has the potential to serve as a core component of a powerful neuropsychological rodent test battery.

## Figures and Tables

**Table 1:** NMDA injection coordinates for hippocampal lesions.

Anterior/posterior	Medial/lateral	Dorsal/ventral	Volume per site (μl)	Post injection diffusion time (min)
−2.8	±1.6	−3.3	0.4	4
−4.2	±2.6	−3	0.4	4
−4.8	±4.8	−6	0.2	2
−5.3	±4.6	−4.2	0.2	2
−5.3	±4.6	−6	0.2	2
−5.8	±4.6	−4.2	0.2	2

**Table 2:** A summary of statistical tested, by experiment, used throughout the study.

Experiment	Statistical tests used	Independent variable	Repeated measure	Repeated measure
1	Repeated measure ANOVA	Lesion	Block (5 days × 4)	
1	Repeated measure ANOVA	Lesion	Separation (3 levels)	
1	SNK post hoc	Lesion	Separation (3 levels)	
2	Repeated measure ANOVA	Lesion	Block (2 days × 4)	
3	Repeated measure ANOVA	Lesion	Delay (1 or 6 s)	Separation (3 levels)
3	Repeated measure ANOVA	Lesion	Delay (1 or 6 s)	
3	Dunnetts *t*-test			
4	Repeated measure ANOVA	Lesion	Separation (6 levels)	
4	SNK post hoc	Lesion	Separation (6 levels)	
5	Bonferoni-correct *t*-test			
5	Non-corrected *t*-test			
